# Fear of hypoglycemia: a key predictor of sleep quality among the diabetic population

**DOI:** 10.3389/fendo.2025.1456641

**Published:** 2025-04-28

**Authors:** Hafiz Rashid Hussain, Nabeel Ahmed, Muhammad Waseem Akram, Faisal Gulzar, Jawad Akbar Khan, Muhammad Asad, Sana Tahseen, Tanveer Ahmed, Abdul Malik, Suhail Akhtar, Ayesha Shahid, Mah Noor, Maryam Pervaiz, Muneeb Ur Rahman

**Affiliations:** ^1^ College of Pharmacy, University of Sargodha, Sargodha, Pakistan; ^2^ School of Economics, University of Wollongong, Wollongong, NSW, Australia; ^3^ Rai Foundation Pharmacy College, Sargodha, Pakistan; ^4^ Riphah Institute of Pharmaceutical Sciences, Riphah International University, Lahore, Pakistan; ^5^ Cadson College of Pharmacy, Kharian, Pakistan; ^6^ Ahmad Polyclinic and Diabetic Center, Sargodha, Pakistan; ^7^ Department of Pharmaceutics, College of Pharmacy, King Saud University, Riyadh, Saudi Arabia; ^8^ Department of Biochemistry, A.T. Still University of Health Sciences, Kirksville, MO, United States

**Keywords:** fear of hypoglycemia, quality of sleep, diabetes mellitus, HFS-II scale, PSQI

## Abstract

**Introduction:**

Every one in seven people with Type-I or Type-II diabetes suffers from fear of hypoglycemia (FOH). Its impact on quality of life, glycemic control, and health outcomes is well studied. However, its relationship with sleep quality remains underexplored, particularly in developing societies. We hypothesize that FOH is a key predictor of sleep quality in Type-I and Type-II patients with diabetes and, therefore, needs detailed investigation.

**Methods:**

A multicentric study was conducted across five cities and six centers of Punjab. Data from 310 diabetes patients were analyzed using the Hypoglycemia Fear Survey-II (HFS-II) Scale and the Pittsburgh Sleep Quality Index (PSQI). Statistical analyses explored subgroup variations, correlations, regression models, and receiver operator curve (ROC) estimation.

**Results:**

The study reports 57.70% of patients with poor sleep among whom 47% had elevated FOH. Sleep quality, age, gender, diabetes duration, and insulin route significantly correlated with FOH (p < 0.05), while glycemic control and insulin use did not. Binary logistics regression showed that for every one-unit increase in FOH, the odds of experiencing poor sleep increased by approximately 3.7% (*p* < 0.001; OR 1.037). Five out of seven sleep components (sleep quality, efficiency, disturbance, medication use, and daytime dysfunction) were significantly related to FOH. We hypothesize that FOH might specifically influence the quality rather than the initiation or termination of the sleep cycle. ROC analysis revealed that HFS-II may be better at diagnosing poor sleep in patients than by chance (p < 0.001) with an AUC of 0.691.

**Conclusion:**

FOH is a key predictor of sleep quality among patients with diabetes. Healthcare providers should prioritize patient education targeting common FOH concerns and assess patients with disturbed sleep for elevated FOH levels, as it may contribute to sleep disturbances.

## 1 Introduction

Diabetes is characterized as an increased blood glucose level that is caused by either a defect in insulin action, insulin secretion, or both, causing various metabolic disorders (1, 2). It is one of the most prevalent diseases across the world with an increasing incidence in Pakistan. The most recent data provided by the International Diabetes Foundation (IDF) has shown a 30.8% prevalence of diabetes in the Pakistani adult population, where every 1 in 4 persons is affected by diabetes (3).

Patients with diabetes are required to maintain a balance between their medication and food intake. This balance is essential to avoid poor glycemic control on one side and hypoglycemia on the other side. Hypoglycemia, although acute, yet is one of the most serious acute adverse outcomes of anti-diabetic treatments (4). It can be manifested as either autonomic symptoms (trembling, palpitations, sweating, tingling) or neuroglycopenic symptoms (difficulty concentrating, confusion, drowsiness, vision changes, difficulty speaking) (5). Generally, the symptoms can be alleviated by the administration of fast-acting carbohydrates and do not require any assistance (level 1 and level 2 hypoglycemia), however, if left unrecognized or unaddressed it may lead to a severe form of hypoglycemia (level 3, requiring aid) resulting in loss of consciousness, seizure, coma, or even death (6). These potentially fatal consequences create a psychological state of mind in diabetes patients known as the fear of hypoglycemia (FOH).

FOH is defined as “the degree of fear associated with episodes of hypoglycemia and their negative consequences” (7). It is one of the most common psychological manifestations associated with hypoglycemia (8–10).

The state of hypoglycemic fear induces behavioral changes in patients with diabetes to prevent the occurrence of hypoglycemic episodes and thus the consequences. These include having additional meals (11), snacking at night (12), stress-induced eating, decreased physical activity (13), maintaining higher blood glucose levels (14, 15), and mismanagement of insulin doses (11, 16–18). These behaviors can potentially lead to impaired glycemic control (10), reduced quality of life (19, 20), suboptimal diabetes management (21, 22), and development of microvascular complications (7, 22).

Along with these physical behaviors, FOH is also manifested psychologically. Evidence shows that FOH is related to anxiety and depression in patients with diabetes (23). These psychological states are closely linked with sleep quality (24, 25). Similarly, research shows that the improvement in blood glucose monitoring technologies has significantly reduced FOH and improved quality of life, including sleep quality, in these patients (26, 27). Therefore, FOH is a potential factor that may affect the quality of sleep in patients with diabetes.

Only a few studies are available worldwide that have assessed this correlation in patients with diabetes (15, 28), but the data on the Pakistani population is scarce. It has also been reported that there is a high prevalence of sleep disturbances among the diabetes population in Pakistan. The results were confirmed in a recent (and by far the first) study by Farooque et al. where 57% of the patients were found to be poor sleepers (Global PSQI Score > 5) in a sample of 329 patients (29). However, the reasons remain unexplored as in the aforementioned study (29), there was no association found between poor sleep and glycemic control which necessitates further research in this area. In light of this, the present study aims to determine if FOH is a key predictor of sleep quality, describe the relationship between FOH and sleep components, and describe factors associated with FOH for targeted interventions.

## 2 Methodology

### 2.1 Study setting and design

This study employed a multicenter cross-sectional research design spanning six months (April 2023 to September 2023). Data was collected from multiple healthcare settings of the Punjab province of Pakistan viz. Ahmad Diabetes and Foot Center, Sargodha; District Headquarter Hospital (DHQ), Sargodha; Non-Communicable Disease Clinic (NCD), DHQ Hospital Jhelum; DHQ Hospital Hafizabad; Tehsil Headquarter Hospital, Lalamusa; and Fatima Memorial Hospital, Sambrial.

### 2.2 Ethics approval

Ethical approval was obtained on April 19, 2023, from the Ethical Review Committee at the University of Sargodha (Reference Number SU/ORIC/799).

### 2.3 Study tools

The study employed two pre-validated questionnaires, viz. the Hypoglycemia Fear Survey (HFS-II) (30) and the Pittsburgh Sleep Quality Index (PSQI) (31). The HFS-II consists of 33 items divided among two sub-scales i.e., the behavior scale (HSF-B, 15 items) and the worry scale (HFS-W, 18 items). It is used to measure different aspects of fear related to hypoglycemia. Each item on the scale carries five Likert points from 0-4 marked as ‘Never’, ‘Rarely’, ‘Sometimes’, ‘Often,’ and ‘Almost always’, respectively. Patients were classified into “elevated fear” or “non-elevated fear” groups based on the elevated item (EI) endorsement criterion (32). The criterion describes patients as having elevated fear if they score ‘≥3’ for more than one item on HFS-W.

The PSQI, with its 19 items, is a widely used tool to assess the sleep quality of patients over the past month based on self-reporting (31). Initial scoring of the scale generates seven components i.e., subjective sleep quality, sleep latency, sleep duration, sleep efficiency, sleep disturbance, medication use, and daytime dysfunction. Each component is scored from 0-3 (0 being the best and 3 being the worst score), and when all seven are cumulated, a global score is generated from ‘0-21’. The patients were categorized into “good sleep health (PSQI ≤ 5)” or “poor sleep health (PSQI > 5)” groups based on the global score (31). Prior permissions were obtained from the relevant bodies/persons to use these questionnaires in this study.

### 2.4 Study participants

A total of 310 participants were enrolled from different centers, as mentioned above. Inclusion criteria comprised patients with Type-I or Type-II diabetes, diabetes duration of ≥ 1 year, good cognitive skills, and willingness to participate. Exclusion criteria included pregnancy-related diabetes, mental illness history, and other conditions hindering communication.

Patients were approached during healthcare visits, and provided with study details, and those agreeing to participate gave written informed consent. Selected patients were then interviewed thoroughly for the baseline demographics, HFS-II, and PSQI questionnaires and responses were marked accordingly.

### 2.5 Statistical analysis

The statistics were applied using the Statistical Package for the Social Sciences (IBM SPSS Statistics for Windows, Version 27.0. Armonk, NY: IBM Corp). Data analysis involved descriptive statistics for means, frequencies, and percentages. The non-normal distribution of dependent variables (HFS-II Total Score and PSQI Global Score) led to the use of non-parametric tests like Kruskal-Wallis and Mann-Whitney tests for significance testing regarding age, gender, education, diabetes duration, and insulin use.

Spearman’s correlation was used to analyze the relationship between fear of hypoglycemia (HFS-II) and sleep quality (PSQI). A binary logistic regression was subsequently performed, treating PSQI as a dichotomous variable for further exploration, with fear of hypoglycemia and insulin use as predictor variables. Finally, ROC curve analysis was conducted to evaluate the diagnostic ability of HFS-II (FOH) in measuring poor sleep quality. The output data on the assumption analysis of regression, normality testing, and ROC curve analysis can be found in the **Supplementary File**.

## 3 Results

### 3.1 Population characteristics

**Table 1** summarizes the population’s characteristics. The largest age group was 41-60 years (30.9%), with 65.5% female and 34.5% male participants. Most had 10 years of education (28.4%), while 11.9% were graduates. Around 40.0% were diagnosed with diabetes in the last 5 years, and 30.0% had diabetes for over 10 years. Mean fasting blood sugar (BSF) was 181.91 mg/dL (SD 70.36), and random blood sugar (BSR) was 268.20 mg/dL (SD 92.17). Notably, 58.4% had poor glycemic control (BSF > 130 mg/dL). Type-I diabetes patients constituted 50% of the sample, primarily using syringes for insulin (41.0%). Additionally, 17.1% used insulin secretagogues and 4.8% used both insulin and insulin secretagogues.

**Table 1:** Sample characteristics of study participants.

Variable	Values	Frequency (%)
Age (Years)	≤ 40	70 (22.6%)
	41-60	190 (61.3%)
	> 60	50 (16.1%)
Gender	Male	107 (34.5%)
	Female	203 (65.5%)
Education	Primary (5 years)	75 (24.2%)
	Middle (8 years)	72 (23.2%)
	Matric (10 years)	88 (28.4%)
	Inter (12 years)	38 (12.3)
	Graduate (14/16 years)	37 (11.9%)
Duration of Diabetes (Years)	< 5	124 (40.0%)
	5 - 10	93 (30.0%)
	> 10	93 (30.0%)
BSF (mg/dL)	Mean	181.91 (SD = 70.364)
BSR (mg/dL)	Mean	268.20 (SD = 92.178)
Glycemic Control	Poor Glycemic Control (FPG > 130 mg/dL)	181 (58.4%)
	Good Glycemic Control (FPG 80 - 130 mg/dL)	60 (19.4%)
	Data Unavailable	69 (22.3%)
Patients on Insulin		155 (50.0%)
Patients on Insulin Secretagogues		53 (17.1%)
Patients on Insulin and Insulin Secretagogue		15 (4.8%)
Method of Insulin Administration	Syringe	127 (41.0%)
	Penfil	45 (14.5%)
	Pump	1 (0.3%)

BSF, Blood Sugar Fasting; BSR, Blood Sugar Random; FPG, Fasting plasma glucose.

### 3.2 HFS-II scores

The mean HFS-II total score was 25.18 (SD 23.24) on a scale of 0 – 132. Mean scores on HFS-B and HFS-W scales were 15.40 (SD 13.64) and 9.77 (SD 12.48) respectively. The most rated items on HFS-B and HFS-W have been listed in **Table 2**. Using the EI criterion (32), we identified 120 patients (38.7%) having an elevated FOH as they scored ‘≥ 3’ for more than one item on HFS-W.

**Table 2:** Most rated items on HFS-II Scale by the study participants.

Scale	Items	Mean (SD)
HFS-B	Stayed at home more than I liked (HFS-B9)	1.55 (1.63)
	Made sure there were other people around me (HFS-B11)	1.50 (1.54)
	Made sure I had someone with me when I went out (HFS-B5)	1.38 (1.50)
	Limited my out-of-town travel (HFS-B6)	1.38 (1.50)
HFS-W	Difficulty thinking clearly (HFS-W12)	1.18 (1.57)
	Feeling lightheaded or dizzy (HFS-W13)	0.83 (1.21)
	No one to help during hypoglycemia (HFS-W8)	0.77 (1.23)
	Becoming upset and difficult (HFS-W18)	0.77 (1.29)

SD, standard deviation; HFS-B, behavior subscale of hypoglycemia fear survey; HFS-W, worry subscale of hypoglycemia fear survey; BG, blood glucose.

### 3.3 FOH in different population groups

The scores of HFS-II, HFS-B, and HFS-W have been presented in **Table 3**. Participant’s gender, diabetes duration, and method of insulin administration were significantly related to the FOH scores. The mean HFS-II score was significantly higher in the female gender (M = 27.40 (SD 22.42)) than in males (M = 20.95 (SD 24.28)). Nearly 75% of the females had an elevated fear of hypoglycemia. The most feared items among females related to hypoglycemia were ‘made sure there were other people around,’ ‘stayed at home more than I liked,’ and ‘made sure I had someone with me when I went out.’ Male participants differed only in item (‘ate large snacks’).

**Table 3:** Fear of hypoglycemia and sleep quality in different population groups.

		The outcome of the HFS-II Total Score			*p*-value^*^	PSQI Outcomes		*p*-value^**^
		Mean Total HFS-II	Mean HFS-B	Mean HFS-W		Good Sleep Health	Poor Sleep Health	
**Age (years)**	≤ 40	26.31 (SD 24.99)	15.58 (SD ± 13.48)	10.73 (SD 13.71)	0.488	29 (41.4%)	41 (58.6%)	0.711
	41-60	24.03 (SD 22.33)	14.58 (SD 13.19)	9.44 (SD 11.94)		80 (42.6%)	110 (57.9%)	
	> 60	27.98 (SD 24.28)	18.30 (SD 15.36)	9.68 (SD 12.89)		22 (44%)	28 (56%)	
**Gender**	Male	20.95 (SD 24.28)	12.69 (SD 14.04)	8.26 (SD 12.44)	**0.002**	49 (45.8%)	58 (54.2%)	0.074
	Female	27.40 (SD 22.42)	16.84 (SD 13.23)	10.56 (SD 12.45)		82 (40.4%)	121 **(59.6%)**	
**Educational Status**	Primary	24.62 (SD 24.21	14.33 (SD 14.38)	10.29 (SD 12.81)	0.881	41 (54.7%)	34 (45.3%)	0.054
	Middle	25.69 (SD 26.28)	15.56 (SD 15.07)	10.12 (SD 14.14)		28 (38.9%)	44 (61.1%)	
	Matric	26.25 (SD 23.16)	17.00 (SD 13.55)	9.25 (SD 11.94)		28 (68.2%)	60 (68.2%)	
	Inter	24.76 (SD 19.98)	14.31 (SD 10.59)	10.44 (SD 12.47)		15 (39.5%)	23 (60.5%)	
	Graduate	23.18 (SD 18.83)	14.62 (SD 12.43)	8.56 (SD 9.82)		19 (51.4%)	18 (48.6%)	
**Duration of Diabetes (years)**	< 5	21.51 (SD 21.43)	13.23 (SD 12.26)	8.28 (SD 11.71)	**0.047**	54 (43.5%)	70 (56.5%)	0.951
	5-10	27.03 (SD 25.31)	16.06 (SD 13.98)	10.96 (SD 14.09)		42 (45.2%)	51 (54.8%)	
	> 10	28.21 (SD 23.01)	17.65 (SD 14.71)	10.55 (SD 11.66)		35 (37.6%)	58 **(62.4%)**	
**Method of Insulin Use**	Syringe	29.40 (SD 25.04)	17.70 (SD 14.68)	11.69 (SD 13.22)	**< 0.001**	57 (44.9%)	70 (55.1%)	0.673
	Penfil	16.17 (SD 18.60)	11.75 (SD 13.13)	4.42 (SD 8.06)		19 (42.2%)	26 **(57.8%)**	
	Pump	37.00	37.00	0.00		1 (100%)	0	
**Glycemic Control**	Poor	22.40 (SD 19.38)	14.34 (SD 12.52)	8.06 (SD 10.27)	0.088	83 (45.9%)	98 (54.1%)	0.137
	Good	30.63 (SD 28.53)	19.01 (SD 15.27)	11.61 (SD 15.46)		21 (35.0%)	39 **(65.0%)**	
**Patients Taking Insulin Only**		26.22 (SD 24.59)	16.77 (SD 14.72)	9.45 (SD 12.52)	0.535	72 (46.5%)	83 **(53.5%)**	**0.047**
**Patients Taking Insulin Secretagogues Only**		21.28 (SD 21.69)	12.52 (SD 12.47)	8.75 (SD 11.61)	0.204	25 (47.2%)	28 (52.8%)	0.959
**Patients Taking Both Medications**		24.46 (SD 20.67)	12.06 (SD 12.80)	12.40 (SD 12.72)	0.752	4 (26.7%)	11 **(73.3%)**	0.237

HFS-II, hypoglycemia fear survey-II; HFS-B, Hypoglycemia fear survey-behavior scale; HFS-W, Hypoglycemia fear survey-worry Scale; PSQI, Pittsburgh Sleep Quality Index; SD, standard deviation; FPG, Fasting plasma glucose.

*Significance was measured between the said variable (age, gender, etc.) and total HFS-II score.

**Significance was measured between the said variable (age, gender, etc.) and the PSQI global score.

Bold values indicate statistical significance, measured as p < 0.05 that we have already mentioned within methodology & results.

Duration of diabetes appears to have a positive correlation with the FOH as the mean HFS-II scores are highest in patients with a duration of > 10 years. Though the overall difference in the groups is marginally significant (*p* = 0.047), the pairwise comparison highlights a more substantial difference of means between the groups ‘< 5 years’ and ‘> 10 years’ (*p* = 0.016).

Finally, the way the patients administer insulin seems to have a significant impact on their FOH. The fear is more prevalent among patients using traditional methods such as insulin syringes (M = 29.40; SD 25.04) than those using modern technologies such as penfills (M = 16.17; SD 18.60). No solid inference can be derived from the scores of patients on insulin pumps due to their small proportion.

Glycemic control (good or bad), insulin *vs* non-insulin users, and the educational status of the participants did not appear to influence the FOH levels significantly. However, the fear was higher in patients with good glycemic control (M = 30.63 (SD 28.53)), use of insulin (M = 26.22 (SD 24.59)), and lower educational level (M = 26.25 (SD 23.16)).

### 3.4 Sleep quality in different population groups

The mean PSQI score was 7.11 (SD 4.31) where 57.7% (179) of the participants were poor sleepers (PSQI > 5) and 42.3% (131) had better sleep (PSQI ≤ 5). Age, gender, diabetes duration, use of oral hypoglycemic agents, glycemic control, and insulin regimen were not related to sleep quality significantly (*p* > 0.05). The use of insulin only, however, had a significant impact on the quality of sleep (*p* = 0.047). The proportion of poor sleep health was higher in all patients with diabetes without regard to their therapeutic regimens i.e., insulin, insulin secretagogues, or both medications. However, sleep health was relatively more compromised in patients using insulins in combination with insulin secretagogues (73.3% poor sleep health). Among other groups, sleep health was more compromised in patients aged between 21-30 years (78.6%), females (59.6%), diabetes duration > 10 years (62.4%), penfil users (57.8%), and those having a good glycemic control (65.0%) (**Table 3**). Interestingly, the FOH also seems to be higher within the same population groups particularly ‘21-30 years old’, ‘females’, ‘diabetes duration >10 years’, and ‘good glycemic control’ (**Table 3**), setting up a base for a more in-depth relation between these two variables explained as follows.

### 3.5 Comparison of FOH and sleep quality

The sleep outcomes were measured as Global PSQI Score (sleep health) and seven sleep components (**Table 4**). A Spearman’s correlation between FOH and sleep health showed a highly significant positive monotonic relation between the two variables (*p* < 0.001, *r*_s_ = 0.397, n = 310). The relationship with behavior and worry dimensions of HFS-II scale was equally significant (*p* < 0.001, *r*_s_ = 0.379; *p* < 0.001, *r*_s_ = 0.304 respectively). Notably, the correlation was positive, meaning the PSQI score increases with the increments in HFS-II scores. Moreover, the mean HFS-II scores were higher among patients with poor sleep health compared to those with good sleep health (**Table 4**). In addition, among the 179 patients with poor sleep health, 46.9% had an elevated FOH according to the EI criterion.

**Table 4:** Relationship between FOH and sleep.

Parameter	Responses	Mean FOH Score	Non-Elevated FOH	Elevated FOH	Significance with FOH*
**PSQI Global**	Good Sleep Health (PSQI ≤ 5)	16.41 (SD 17.07)	95 (72.5%)	36 (27.5%)	**< 0.001**
	Poor Sleep Health (PSQI > 5)	31.59 (SD 25.04)	95 (53.1%)	84 (46.9%)	
**Subjective Sleep Quality**	Very Good	21.14 (SD 19.68)	73 (67.0%)	36 (33.0%)	**0.007**
	Fairly Good	22.85 (SD 22.35)	63 (63.6%)	36 (36.4%)	
	Fairly Bad	32.96 (SD 27.18)	27 (51.9%)	25 (48.1%)	
	Very Bad	30.48 (SD 25.39)	27 (54.0%)	23 (46.0%)	
**Latency**	0	23.03 (SD 22.69)	69 (63.9%)	39 (36.1%)	0.145
	1-2	23.85 (SD 23.20)	50 (64.1%)	28 (35.9%)	
	3-4	25.04 (SD 21.24)	42 (60.0%)	28 (40.0%)	
	5-6	31.55 (SD 26.23)	29 (53.7%)	25 (46.3%)	
**Duration**	> 7 hours	25.07 (SD 23.76)	87 (62.1%)	53 (37.9%)	0.626
	6-7 hours	24.35 (SD 23.32)	44 (56.4%)	34 (43.6%)	
	5-6 hours	23.80 (SD 23.22)	27 (67.5%)	13 (32.5%)	
	< 5 hours	27.75 (SD 22.18)	32 (61.5%)	20 (38.5%)	
**Efficiency**	> 85%	23.03 (SD 23.50)	133 (65.2%)	71 (34.8%)	**0.017**
	75-84%	26.29 (SD 22.50)	17 (63.0%)	10 (37.0%)	
	65-74%	19.57 (SD 20.67)	6 (85.7%)	1 (14.3%)	
	< 65%	31.38 (SD 22.24)	34 (47.2%)	38 (52.8%)	
**Disturbance**	0	7.07 (SD 7.64)	13 (100%)	0 (0%)	**< 0.001**
	1 - 9	16.95 (SD 18.66)	110 (73.8%)	39 (26.2%)	
	10 - 18	31.97 (SD 22.57)	60 (49.6%)	61 (50.4%)	
	19 - 27	48.85 (SD 26.68)	7 (25.9%)	20 (74.1%)	
**Medication**	Not during the past month	20.96 (SD 19.99)	149 (65.1%)	80 (34.9%)	**< 0.001**
	Less than once a week	23.88 (SD 18.55)	23 (65.7%)	12 (34.3%)	
	Once or twice a week	46.76 (SD 27.15)	12 (46.2%)	14 (53.8%)	
	Three or more times a week	47.70 (SD 31.85)	6 (30.0%)	14 (70.0%)	
**Day Time Dysfunction**	0	15.95 (SD 17.53)	96 (74.4%)	33 (25.6%)	**< 0.001**
	1 - 2	23.05 (SD 20.13)	53 (61.6%)	33 (38.4%)	
	3 - 4	38.14 (SD 22.96)	33 (47.1%)	37 (52.9%)	
	5 - 6	43.80 (SD 32.25)	8 (32.0%)	17 (68.0%)	

*The significance was measured between the categorical sleep components and HFS-II total scores.

Bold values indicate statistical significance, measured as p < 0.05 that we have already mentioned within methodology & results.

Going ahead, we attempted to understand the relationship between FOH and the sleep components to highlight grey areas in overall sleep health. The relationship of five out of seven components was significant *(p < 0.05)*. These were subjective sleep quality, sleep efficiency, sleep disturbance, sleep medications, and daytime dysfunction. Sleep latency and duration showed an insignificant relationship with FOH. With few exceptions, the mean FOH scores as well as the proportion of elevated fear tend to rise with the worsening outcome of each sleep component. For example, a ‘fairly bad’ subjective sleep quality in patients is associated with 48.1% elevated FOH compared to 36.4% for ‘fairly good’ sleep quality. A similar trend is followed in other components except for the sleep duration where a relatively large deviation can be seen.

### 3.6 Relationship between FOH and sleep quality

To further understand the nature of the relationship between FOH and sleep quality, logistic regression analysis was performed between the two variables (**Table 5**). The global PSQI score (continuous scale, 0-21) was converted into a binary variable ‘sleep health’ with two outputs, good sleep health (PSQI ≤ 5) and poor sleep health (PSQI > 5), to perform a binary logistic regression. The ‘HFS-II score’ (continuous variable) was added as the independent variable with ‘insulin use (dichotomous)’ as a covariate since it was found to be significantly related to sleep quality (**Table 3**). The model demonstrated an overall satisfactory fit, as evidenced by the -2 Log Likelihood value of 382.370 and the Cox & Snell R Square (12.1%) and Nagelkerke R Square (16.2%). For the predictor variable “Fear of hypoglycemia,” the coefficient (B) was 0.036, and the Wald chi-square test yielded a statistically significant result (χ² (1) = 29.387, *p* < 0.001). The odds ratio (Exp(B)) was 1.037, suggesting that for every one-unit increase in fear of hypoglycemia, the odds of experiencing poor sleep increased by approximately 3.7%. As for “Insulin use,” the coefficient (B) was -0.440, and the Wald chi-square test produced a *p*-value of 0.073, indicating marginal significance. The odds ratio (Exp(B)) for insulin use was 0.644, suggesting that individuals using insulin had approximately 60% lower odds of experiencing poor sleep compared to those not using insulin.

**Table 5:** Logistic regression predicting sleep health from FOH and insulin use.

Predictor	*B*	Wald χ*^2^*	*p*	Odds Ratio	95% CI for EXP(B)	
					Lower	Upper
Total HFS Score	0.036	29.387	< 0.001	1.037	1.023	1.050
Patients Taking Insulin Only	-0.440	3.212	0.073	0.644	0.398	1.042
Constant	-0.293	1.845	0.174	0.746		

### 3.7 Diagnostic ability of FOH to predict poor sleep quality – receiver operating characteristic (ROC) curve analysis

**Figure 1:**
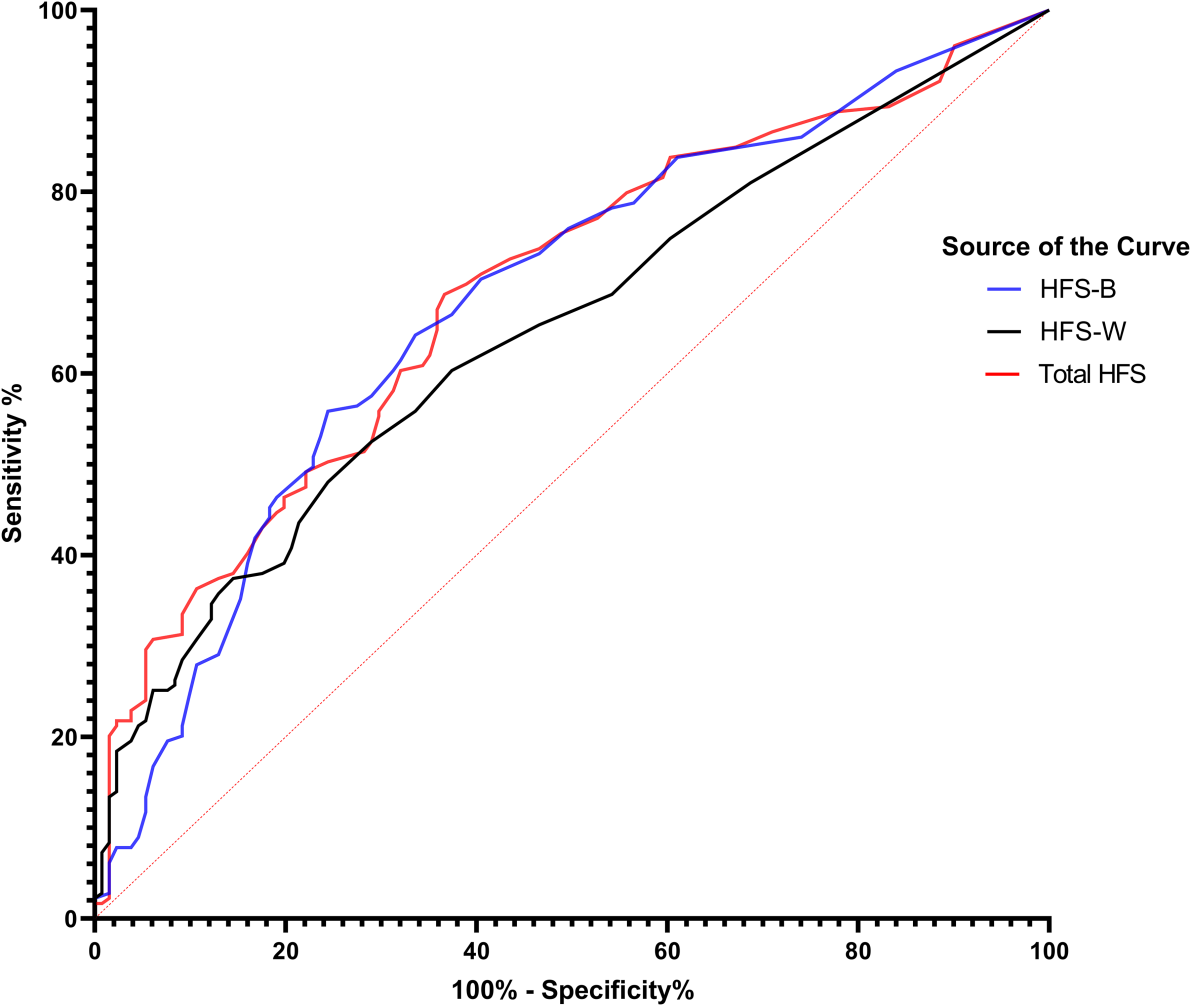
ROC Curves of fear of hypoglycemia scores predicting sleep quality in patients with diabetes. This figure shows ROC curves for components of the Fear of Hypoglycemia Survey (HFS) in predicting sleep quality among patients with diabetes. The blue curve (HFS-B) represents the behavioral component, the black curve (HFS-W) represents the worry component, and the red curve (Total HFS) represents the combined score. The diagonal red dashed line indicates no discrimination (AUC = 0.5). Higher AUC values denote better predictive accuracy.

ROC curve analysis is a graphical representation used to evaluate the diagnostic ability of a binary classifier system. In the present case, the ROC curve analysis was conducted to evaluate the diagnostic accuracy of three scales—HFS-B (Behavior score), HFS-W (Worry score), and HFS-T (Total HFS score)—in identifying poor sleep quality (**Table 6**, **Figure 1**). The dataset comprised 179 cases with severe sleep difficulty and 131 cases without. The Area Under the Curve (AUC) values were 0.691 for the Total HFS Score, 0.681 for the HFS-B Scores, and 0.647 for the HFS-W Scores, with all p-values being < 0.001. This indicates that all three scales are significantly better than chance at distinguishing between individuals with and without severe sleep difficulty. Following this, we analyzed sensitivity and 1 – specificity values of the three scales at a cutoff value of 3.5. The total HFS-II score showed the highest sensitivity, followed by HFS-B and HFS-W. However, as a trade-off between sensitivity and specificity, HFS-B appears to be more optimal with a specificity of 83.8% and a false-positive rate of 61.1%.

**Table 6:** Diagnostic performance of Total HFS, HFS-B, and HFS-W scores for identifying poor sleep quality using ROC.

Test Result Variable	AUC	Std. Error	p-value	95% CI (Lower)	95% CI (Upper)	Cutoff	Sensitivity	1 - Specificity
Total HFS Score	0.691	0.030	0.000	0.632	0.749	3.5000	0.888	0.779
HFS-B Scores	0.681	0.031	0.000	0.621	0.741	3.5000	0.838	0.611
HFS-W Scores	0.647	0.031	0.000	0.587	0.708	3.5000	0.654	0.466

## 4 Discussion

This is the first study examining the FOH among patients with diabetes and its influence on the quality of sleep in Pakistan. While the clinical practice is intensely oriented to reducing glycemic levels, the resultant hypoglycemic events and associated psychological implications are often ignored. FOH is one of the most common psychological manifestations of hypoglycemia. It is known to impact behavioral and worry patterns in individuals with diabetes. However, its impact on psychological states, such as sleep, is still in its infancy. Few studies that examined this particular relationship were related to Type-I patients (28) or adolescents (33).

In our study, with the inclusion of both Type-I and Type-II diabetics and no age restriction, the mean HFS-II score is 25.18 (SD 23.24) which is relatively lower compared to similar studies measuring FOH (10, 30, 34, 35). However, like our study, some other studies have also reported lower mean values for HFS scores (36). The EI criterion classified 120 individuals (38.7%) as having an elevated FOH which is relatively higher than other studies by Hajos et al. (32) (26%) and Majanovic et al. (37) (11.1%). The mean differences as well as fear characteristics could be attributed to the population differences in these studies.

In our study, FOH was higher in younger age (<10 – 30 years) as supported by other studies (35, 38, 39). Also, higher FOH was seen in patients above 60 years which is also evident from the literature (10, 11). We have also noted that the most vulnerable age groups with respect to FOH are those below 40 years and above 50 years. However, we agree with the interpretation of Martyn-Nemet et al. (40) that there is no consistent pattern of FOH with regard to age and that the relationship with age is complex. For gender comparisons, FOH was higher in females which are in uniform agreement with the previous literature (38, 41, 42).

Education seems to have a positive impact on the FOH. The fear is higher among participants with lower educational levels and vice versa. This appears to be a negative correlation as reported by Gonder-Fredrick et al. (30), however, we could not perform a direct correlation statistic due to the categorical nature of our variable. The relationship, nonetheless, remains insignificant and consistent with other studies (41).

Duration of diabetes was significantly associated with FOH in our study. The scores of HFS-II increased with the increase in the duration of diabetes. The findings are consistent with previous studies by Hongmei et al. (43) and Erol and Enc (41) where a positive correlation existed between the course of disease (diabetes) and FOH. Opposite findings also surfaced in some populations where there was no correlation between duration of diabetes and FOH or the FOH decreased with an increase in the duration of diabetes (30, 44).

The impact of glycemic control on FOH is debated. Some studies suggest improved glycemic control is associated with increased FOH (35, 40, 45), while others show higher FOH in patients with poor control (36). In our study, glycemic control showed a marginally insignificant relationship with FOH. However, patients with good control (FPG 80-130 mg/dL) were more likely to have FOH than those with poorer control (FPG > 130 mg/dL). Tight control lowers glucose levels intensively, raising hypoglycemia risk and concerns. Regular glucose monitoring and insulin dose management are key, especially in T2DM.

The FOH did not differ significantly among insulin users and those on oral anti-diabetic agents. However, the proportion of FOH was greater among insulin users compared to other groups. Studies have found a similar pattern of FOH among Type-I and Type-II diabetes patients, that is, individuals with T1DM have more FOH than individuals with T2DM, but the difference is not statistically significant (35, 36, 41).

As for the technology used for insulin administration, our data suggested that FOH was more prevalent in those administering insulin via syringes than those using pen fills. This means that technology infuses confidence and security among patients and is a reliable tool for reducing FOH. These results are consistent with a previous study showing that FOH was higher in patients using multiple-dose injection treatment compared to those receiving insulin via pump (38). However, in our study patients on insulin pumps showed a higher proportion of FOH but given the small number of users, the results cannot be generalized.

The relationship between FOH and sleep appears to be fairly significant. The univariate analysis as well as the multivariate logistic regression found the relationship significant. Moreover, five out of seven PSQI sleep components showed a statistically significant link with FOH. Sleep latency and sleep duration were not significantly linked with FOH. This generates the hypothesis that the FOH may not affect the initiation or termination of the sleep cycle rather it is more specifically linked with the quality of sleep. This could be disturbed sleep (*p* < 0.001*)*, less efficient sleep (*p* = 0.017), medication-dependent sleep (*p* < 0.001), or sleep leading to daytime dysfunction (*p* < 0.001). These findings represent a novel contribution to the existing literature, signaling a need for further exploration and in-depth investigation into the specific mechanisms through which FOH manifests its impact on the qualitative aspects of sleep.

The ROC curve estimation also provided some novel insights into utilization of HFS-II scores in determining the quality of sleep. The results revealed that the HFS scores were better at predicting sleep quality than by chance. However, we couldn’t determine a specific cut-off score for a reliable diagnosis as the false positive rates were high. For the sake of comparison, a cut-off value of 3.5 was selected, which was closest to the maximum possible score on one item. This approach showed that the HFS-B scale was better at predicting poor sleep quality than the other forms of the scale in terms of sensitivity and specificity. This could be because of the underlying condition that the means of the HFS-B scores were higher in our study participants (15.40 (SD 13.64)) compared to the HFS-W scores (9.77 (SD 12.48)) indicating that fear derived from behavioral aspects may be more limiting towards sleep quality. However, the findings need clinical support to reflect more authenticity.

The identification of these nuanced relationships provides a foundation for targeted interventions aimed at improving sleep quality in individuals grappling with the psychological challenges associated with FOH. As the field advances, this newfound understanding paves the way for tailored strategies that encompass both glycemic control and psychological well-being in the holistic care of patients with diabetes. Further research endeavors should delve into the intricate dynamics of FOH and its implications on sleep, contributing to the evolving landscape of diabetes management and patient-centric care.

The strength of this study is that, for the first time, it provides comprehensive data on FOH in the diabetes population of Pakistan. While most of the studies utilize only the worry scale of HFS-II, we used the complete 33-item HFS-II scale in our sample population. Another strength of the study is that patients with both T1DM and T2DM were included in this study, unlike previous studies where the choice was selective. Finally, the study outlines an in-depth association between FOH and sleep quality and evaluates the diagnostic ability of FOH in terms of sleep quality. Moreover, it proposes a new hypothesis that the FOH may influence not the initiation and termination of the sleep cycle but its quality in an individual.

## 5 Conclusion

In conclusion, this study sheds light on the underexplored issue of FOH in the Pakistani population with diabetes, revealing its significant impact on both psychological well-being and sleep quality. The findings underscore the prevalence of FOH, with particular vulnerability among younger and older age groups, females, syringe users, and those with longer diabetes durations. Notably, FOH is closely linked with poor sleep quality, emphasizing the need for holistic diabetes management that addresses both glycemic control and psychological aspects. Healthcare practitioners should prioritize patient education and counseling, targeting the most common FOH concerns identified in this study. Furthermore, patients with disturbed sleep should be assessed for an elevated level of FOH as it could be a contributing factor to poor sleep quality. Future research should develop validated methods to better understand the intricate relationship between FOH and sleep disturbances.

## 6 Limitations

The cross-sectional design, missing confounding variables (e.g., history of hypoglycemic episodes, comorbidities e.g., obesity), and a higher proportion of female participants are some of the limitations of this study. Moreover, the data on glycemic control was generated from a single value of either BSR or BSF, which may not reflect the chronic condition of glycemic control. HbA1c could have been a better measure; however, we could not find enough data for this. Additionally, the PSQI tool assesses sleep quality over a relatively short period (past month) and may not capture long-term sleep patterns accurately. Furthermore, using HFS-II scores alone to classify patients into “elevated fear” or “non-elevated fear” groups may oversimplify FOH, which varies in intensity and impact among individuals. Similarly, future studies are also recommended to explore the relationship between fear of hypoglycemia and sleep quality in patients on non-hypoglycemic therapies.

## Data Availability

The original contributions presented in the study are included in the article/**Supplementary Material**. Further inquiries can be directed to the corresponding authors.
