# Supplementary material for: Fear of hypoglycemia: a key predictor of sleep quality among the diabetic population
Source: Front Endocrinol (Lausanne). 2025 Apr 28;16:1456641. doi: 10.3389/fendo.2025.1456641 (PMC12066330; doi:10.3389/fendo.2025.1456641)
Supplement: Supplementary file 1 [file DataSheet1.docx]

Supplementary Material

# Distributions of the dependent variables

The distributions for the dependent variables i.e., fear of hypoglycemia (HFS-II scores) and sleep quality (PSQI global scores) were checked and found to be non-normally distributed.

**Supplementary Table 1**. Test of normality of PSQI and HFS-II scores

| **Tests of Normality** | | | | | | |
| --- | --- | --- | --- | --- | --- | --- |
|  | Kolmogorov-Smirnov^a^ | | | **Shapiro-Wilk** | | |
|  | Statistic | df | Sig. | Statistic | df | Sig. |
| PSQI Global Score | .110 | 310 | .000 | .955 | 310 | .000 |
| Total HFS Score | .139 | 310 | .000 | .885 | 310 | .000 |
| a. Lilliefors Significance Correction | | | | | | |


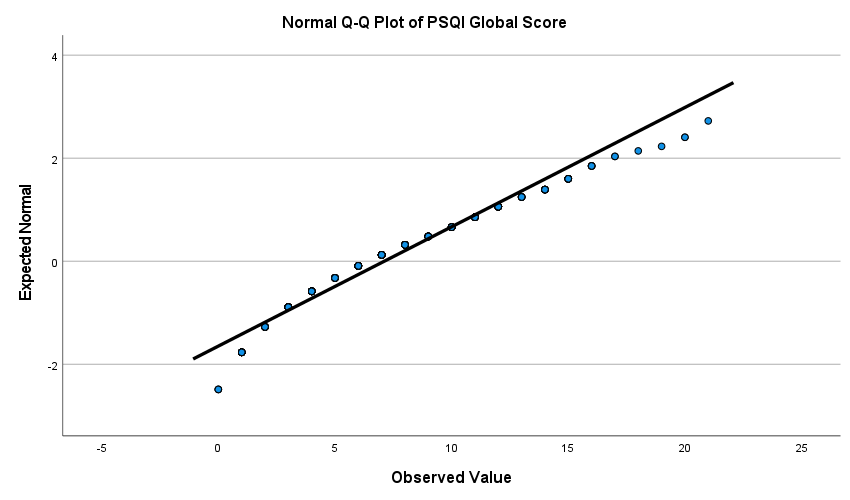


**Supplementary Figure 1.** Q-Q plot of PSQI global scores


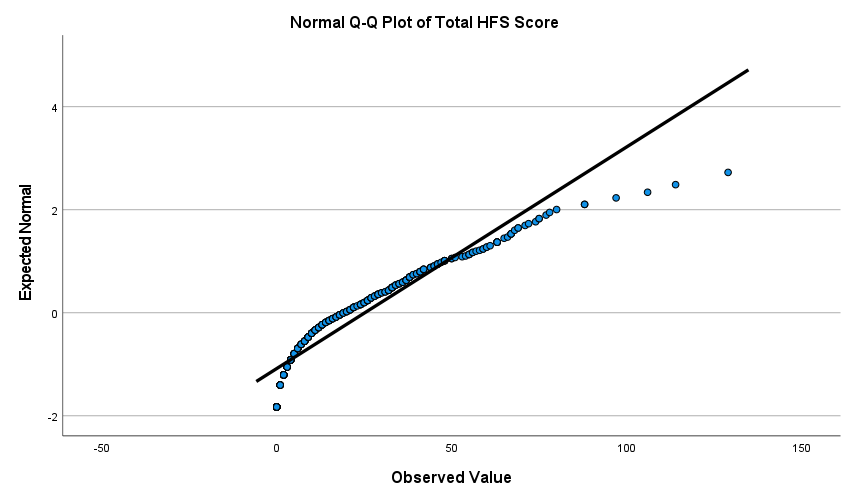


**Supplementary Figure 2.** Normal Q-Q plot of total HFS-II scores

# Assumption analysis for the binary logistic regression predicting the outcome of sleep quality with FOH and use of insulin

**Assumption 1: Binary Dependent Variable**

The dependent variable was ‘Sleep outcome’ coded as ‘0’ for good sleep (PSQI ≤ 5) and ‘1’ for poor sleep or severe sleep difficulty in at least 2 areas (PSQI > 5).

**Assumption 2: Independent Observations**

The study design ensured that each observation was independent of the others.

**Assumption 3: No Multicollinearity**

The assumption of multicollinearity was checked through a correlation matrix and variance inflation factor (VIF). Correlations above 0.8 or below -0.8 may indicate multicollinearity. VIF values above 10 indicate serious multicollinearity issues. The correlation matrix is shown below with a value of 0.035.

**Supplementary Table 2.** Correlation matrix of predictors to check multicollinearity

| **Correlations** | | | | |
| --- | --- | --- | --- | --- |
|  | | | **Patients Taking Insulin Only** | **Total HFS Score** |
| **Spearman's rho** | **Patients Taking Insulin Only** | Correlation Coefficient | 1.000 | .035 |
|  |  | Sig. (2-tailed) | . | .536 |
|  |  | N | 310 | 310 |
|  | **Total HFS Score** | Correlation Coefficient | .035 | 1.000 |
|  |  | Sig. (2-tailed) | .536 | . |
|  |  | N | 310 | 310 |

**Assumption 4: Linearity of Logit**

The continuous predictor should have linear relationship with the logit of the outcome. Using Box-Tidwell Test, an interaction term was created and checked for significance. The term was found to be non-significant (p = 0.754) indicating linear relationship.

**Supplementary Table 3.** Regression analysis with interaction term to exclude non-linearity

| **Variables in the Equation** | | | | | | | |
| --- | --- | --- | --- | --- | --- | --- | --- |
|  | | **B** | **S.E.** | **Wald** | **df** | **Sig.** | **Exp(B)** |
| Step 1^a^ | **Total HFS Score** | .054 | 0.058 | .876 | 1 | .349 | 1.055 |
|  | **Patients Taking Insulin Only** | -.442 | 0.246 | 3.224 | 1 | .073 | .643 |
|  | **Total_HFS_LN by Total HFS Score** | -.004 | .014 | .098 | 1 | .754 | .996 |
|  | **Constant** | -.357 | .299 | 1.424 | 1 | .233 | .700 |
| a. Variable(s) entered on step 1: Total HFS Score, Patients Taking Insulin Only, Total_HFS_LN * Total HFS Score. | | | | | | | |

**Assumption 5: Absence of Outliers**

Outliers were detected using standardized residuals. Standardized residuals greater than 3 or less than -3 have the potential to be outliers. Two observations (146, 178) had standardized values of -3.38511 and -3.98119 respectively. The regression was performed with and without these potential outliers. The predicted responses, estimated slope coefficients, and hypothesis test results were not affected by the inclusion of these data points. Therefore, the data points were not deemed influential

**Assumption 6: Adequate Sample Size**

A common rule of thumb is at least 10 events per predictor variable.

**Supplementary Table 4.** Crosstab results between the categorical variable (Insulin use) and the binary outcome to check for adequate sample size

|  | | **Overall Sleep Quality** | | **Total** |
| --- | --- | --- | --- | --- |
|  |  | **Good Sleep** | **Severe Sleep Difficulty in at least 2 areas (PSQI > 5)** |  |
| **Patients Taking Insulin Only** | No | 59 | 96 | 155 |
|  | Yes | 72 | 83 | 155 |
| **Total** | | 131 | 179 | 310 |

**Assumption 7: Model Fit**

Model fit was checked through the Hosmer-Lemeshow Test. A non-significant p-value was obtained indicating a good fit.

**Supplementary Table 5.** Hosmer and Lemeshow test to measure model fit

| **Step** | **Chi-square** | **df** | **Sig.** |
| --- | --- | --- | --- |
| 1 | 6.863 | 8 | .551 |

# ROC Curve Analysis

**Supplementary Table 6**. Case processing summary for ROC curve analysis

| Overall Sleep Quality^a^ | Valid N (listwise) |
| --- | --- |
| Positive^b^ | 179 |
| Negative | 131 |
| Larger values of the test result variable(s) indicate stronger evidence for a positive actual state. | |
| a. The test result variable(s): HFS-W Scores has at least one tie between the positive actual state group and the negative actual state group. | |
| b. The positive actual state is Severe Sleep Difficulty in at least 2 areas (PSQI > 5). | |

**Supplementary Table 7**. Area under the curve values for diagnostic scales (HFS-II, HFS-B, HFS-W) with asymptotic significance and confidence intervals.

| **Test Result Variable(s)** | **Area** | **Std. Error^a^** | **Asymptotic Sig.^b^** | **Asymptotic 95% Confidence Interval** | |
| --- | --- | --- | --- | --- | --- |
|  |  |  |  | **Lower Bound** | **Upper Bound** |
| Total HFS Score | .691 | .030 | .000 | .632 | .749 |
| HFS-B Scores | .681 | .031 | .000 | .621 | .741 |
| HFS-W Scores | .647 | .031 | .000 | .587 | .708 |
| The test result variable(s): Total HFS Score, HFS-B Scores, HFS-W Scores has at least one tie between the positive actual state group and the negative actual state group. Statistics may be biased. | | | | | |
| a. Under the nonparametric assumption | | | | | |
| b. Null hypothesis: true area = 0.5 | | | | | |

**Supplementary Table 8**. Sensitivity and false-positive rates for different cut-off values of the FOH scales

| **Test Result Variable(s)** | **Positive if Greater Than or Equal To^a^** | **Sensitivity** | **1 - Specificity** |
| --- | --- | --- | --- |
| **Total HFS Score** | -1.0000 | 1.000 | 1.000 |
|  | .5000 | .961 | .901 |
|  | 1.5000 | .922 | .885 |
|  | 2.5000 | .894 | .832 |
|  | 3.5000 | .888 | .779 |
|  | 4.5000 | .866 | .710 |
|  | 5.5000 | .849 | .672 |
|  | 6.5000 | .838 | .603 |
|  | 7.5000 | .816 | .595 |
|  | 8.5000 | .799 | .557 |
|  | 9.5000 | .771 | .527 |
|  | 10.5000 | .754 | .489 |
|  | 11.5000 | .737 | .466 |
|  | 12.5000 | .726 | .435 |
|  | 13.5000 | .709 | .405 |
|  | 14.5000 | .698 | .389 |
|  | 15.5000 | .687 | .366 |
|  | 16.5000 | .670 | .359 |
|  | 17.5000 | .648 | .359 |
|  | 18.5000 | .620 | .351 |
|  | 19.5000 | .609 | .344 |
|  | 20.5000 | .603 | .321 |
|  | 21.5000 | .581 | .313 |
|  | 22.5000 | .559 | .298 |
|  | 23.5000 | .553 | .298 |
|  | 24.5000 | .525 | .290 |
|  | 25.5000 | .514 | .282 |
|  | 26.5000 | .503 | .244 |
|  | 27.5000 | .492 | .221 |
|  | 28.5000 | .475 | .221 |
|  | 29.5000 | .464 | .198 |
|  | 30.5000 | .453 | .198 |
|  | 31.5000 | .447 | .191 |
|  | 32.5000 | .430 | .176 |
|  | 33.5000 | .402 | .160 |
|  | 34.5000 | .391 | .153 |
|  | 35.5000 | .380 | .145 |
|  | 36.5000 | .374 | .130 |
|  | 37.5000 | .363 | .107 |
|  | 38.5000 | .335 | .092 |
|  | 39.5000 | .324 | .092 |
|  | 40.5000 | .313 | .092 |
|  | 41.5000 | .307 | .061 |
|  | 43.0000 | .296 | .053 |
|  | 44.5000 | .279 | .053 |
|  | 45.5000 | .268 | .053 |
|  | 46.5000 | .246 | .053 |
|  | 47.5000 | .240 | .053 |
|  | 49.0000 | .229 | .038 |
|  | 50.5000 | .218 | .038 |
|  | 52.0000 | .218 | .031 |
|  | 53.5000 | .218 | .023 |
|  | 54.5000 | .212 | .023 |
|  | 55.5000 | .201 | .015 |
|  | 56.5000 | .190 | .015 |
|  | 57.5000 | .184 | .015 |
|  | 58.5000 | .179 | .015 |
|  | 59.5000 | .168 | .015 |
|  | 60.5000 | .156 | .015 |
|  | 62.0000 | .151 | .015 |
|  | 64.0000 | .117 | .015 |
|  | 65.5000 | .112 | .015 |
|  | 66.5000 | .106 | .015 |
|  | 67.5000 | .084 | .015 |
|  | 68.5000 | .078 | .015 |
|  | 70.0000 | .067 | .015 |
|  | 71.5000 | .061 | .015 |
|  | 73.0000 | .056 | .015 |
|  | 74.5000 | .050 | .015 |
|  | 76.0000 | .039 | .015 |
|  | 77.5000 | .034 | .015 |
|  | 79.0000 | .028 | .015 |
|  | 84.0000 | .022 | .015 |
|  | 92.5000 | .017 | .008 |
|  | 101.5000 | .017 | .000 |
|  | 110.0000 | .011 | .000 |
|  | 121.5000 | .006 | .000 |
|  | 130.0000 | .000 | .000 |
| **HFS-B Scores** | -1.0000 | 1.000 | 1.000 |
|  | .5000 | .933 | .840 |
|  | 1.5000 | .888 | .779 |
|  | 2.5000 | .860 | .740 |
|  | 3.5000 | .838 | .611 |
|  | 4.5000 | .788 | .565 |
|  | 5.5000 | .782 | .542 |
|  | 6.5000 | .760 | .496 |
|  | 7.5000 | .732 | .466 |
|  | 8.5000 | .704 | .405 |
|  | 9.5000 | .665 | .374 |
|  | 10.5000 | .642 | .336 |
|  | 11.5000 | .615 | .321 |
|  | 12.5000 | .603 | .313 |
|  | 13.5000 | .575 | .290 |
|  | 14.5000 | .564 | .275 |
|  | 15.5000 | .559 | .244 |
|  | 16.5000 | .531 | .237 |
|  | 17.5000 | .508 | .229 |
|  | 18.5000 | .497 | .229 |
|  | 19.5000 | .492 | .221 |
|  | 20.5000 | .464 | .191 |
|  | 21.5000 | .453 | .183 |
|  | 22.5000 | .441 | .183 |
|  | 23.5000 | .419 | .168 |
|  | 24.5000 | .391 | .160 |
|  | 25.5000 | .352 | .153 |
|  | 26.5000 | .291 | .130 |
|  | 27.5000 | .279 | .107 |
|  | 28.5000 | .246 | .099 |
|  | 29.5000 | .212 | .092 |
|  | 30.5000 | .201 | .092 |
|  | 31.5000 | .196 | .076 |
|  | 32.5000 | .168 | .061 |
|  | 33.5000 | .134 | .053 |
|  | 34.5000 | .117 | .053 |
|  | 35.5000 | .089 | .046 |
|  | 36.5000 | .078 | .038 |
|  | 38.0000 | .078 | .023 |
|  | 40.5000 | .061 | .015 |
|  | 43.0000 | .056 | .015 |
|  | 45.5000 | .045 | .015 |
|  | 48.0000 | .039 | .015 |
|  | 50.0000 | .028 | .015 |
|  | 53.0000 | .022 | .000 |
|  | 56.5000 | .011 | .000 |
|  | 59.0000 | .000 | .000 |
| **HFS-W Scores** | -1.0000 | 1.000 | 1.000 |
|  | .5000 | .810 | .687 |
|  | 1.5000 | .749 | .603 |
|  | 2.5000 | .687 | .542 |
|  | 3.5000 | .654 | .466 |
|  | 4.5000 | .603 | .374 |
|  | 5.5000 | .559 | .336 |
|  | 6.5000 | .525 | .290 |
|  | 7.5000 | .480 | .244 |
|  | 8.5000 | .436 | .214 |
|  | 9.5000 | .408 | .206 |
|  | 10.5000 | .391 | .198 |
|  | 11.5000 | .380 | .176 |
|  | 12.5000 | .374 | .145 |
|  | 13.5000 | .358 | .130 |
|  | 14.5000 | .346 | .122 |
|  | 15.5000 | .330 | .122 |
|  | 16.5000 | .307 | .107 |
|  | 17.5000 | .285 | .092 |
|  | 18.5000 | .263 | .084 |
|  | 19.5000 | .257 | .084 |
|  | 20.5000 | .251 | .076 |
|  | 21.5000 | .251 | .061 |
|  | 22.5000 | .218 | .053 |
|  | 23.5000 | .212 | .046 |
|  | 24.5000 | .196 | .038 |
|  | 25.5000 | .184 | .023 |
|  | 26.5000 | .173 | .023 |
|  | 27.5000 | .168 | .023 |
|  | 28.5000 | .156 | .023 |
|  | 29.5000 | .151 | .023 |
|  | 30.5000 | .140 | .023 |
|  | 31.5000 | .134 | .015 |
|  | 32.5000 | .123 | .015 |
|  | 33.5000 | .117 | .015 |
|  | 34.5000 | .112 | .015 |
|  | 35.5000 | .089 | .015 |
|  | 36.5000 | .084 | .015 |
|  | 38.0000 | .073 | .008 |
|  | 39.5000 | .067 | .008 |
|  | 41.0000 | .061 | .008 |
|  | 42.5000 | .045 | .008 |
|  | 43.5000 | .039 | .008 |
|  | 44.5000 | .034 | .008 |
|  | 45.5000 | .028 | .008 |
|  | 47.5000 | .022 | .000 |
|  | 50.0000 | .017 | .000 |
|  | 53.5000 | .011 | .000 |
|  | 63.5000 | .006 | .000 |
|  | 72.0000 | .000 | .000 |
| **The test result variable(s): Total HFS Score, HFS-B Scores, HFS-W Scores has at least one tie between the positive actual state group and the negative actual state group.** | | | |
| **a. The smallest cutoff value is the minimum observed test value minus 1, and the largest cutoff value is the maximum observed test value plus 1. All the other cutoff values are the averages of two consecutive ordered observed test values.** | | | |
